# Keystone Veterinary Medical Association

**Published:** 1894-01

**Authors:** Walter L. Hart

**Affiliations:** Secretary


### KEYSTONE VETERINARY MEDICAL ASSOCIATION.

The regular monthly meeting of the Keystone Veterinary Medical Association was held Tuesday evening, December 12th, 1893.

The meeting was called to order by the President, Dr. Chas. T. Gœntner; there answered roll call Drs. J. B. Raynor, Hoskins, W. D. Hart, J. R. Hart and Gœntner.

The minutes of the previous meeting were read and approved. The Board of Censors for the ensuing year, Drs. James B. Raynor, W. Horace Hoskins, and Walter L. Hart were appointed. The Board Censors ordered at the last meeting to look after the Prothonotary's office to make necessary changes in their mode of registering, reported through Dr. Hoskins that they had interviewed the registering clerk who has charge of the veterinary register as to the changes that had occurred in the profession, and notified him that they would give him the dates of all the changes as they shall occur, so that he shall be able to keep proper record of the same.

It was moved and seconded that Dr. H. B. Felton's resignation be laid over until next meeting, so that the Secretary can look over the minutes of the previous meeting to see if it had been acted on before.

The Board of Censors reported favorably the applications of Drs. W. L. Rhoads and H. J. McClellan, of Lansdowne, Pa. (American Veterinary College), and they were elected members of the Association unanimously.

Dr. W. S. Hooker was elected delegate to represent the Association at the meeting of the New Jersey Veterinary Medical Association to be held at Morristown, N. J., December 14th, '93.

It was moved by Dr. Hoskins, seconded by Dr. Gœntner, that the Association request their Board of Censors to draw up suitable resolutions approving that part of the President of the United States message that relates to the merit system in the appointment of Veterinary Surgeon in the Bureau of Animal Industry, and to his efforts to stamp out contagious disease in cattle.

Adjourned to meet second Tuesday in January, 1894.

WALTER L. HART, D.V.S., *Secretary.*

---

